# Recurrent myelitis secondary to peripheral nerve root stretching: A case report and review of the literature

**DOI:** 10.1016/j.tcr.2025.101178

**Published:** 2025-04-15

**Authors:** Abbas Rahimi Jaberi, Ghazanfar Rafiee

**Affiliations:** a Neurology Department, Medical School, Shiraz University of Medical Sciences, Shiraz, Iran; b Medical-Surgical Nursing Department, Shiraz University of Medical Sciences, Shiraz, Iran

**Keywords:** Stretch, Myelitis, Peripheral nerve root, Case report

## Abstract

Stretching a nerve more than 5% of its resting length for an extended period can result within changes dysfunction and ischemia. Nerve damage known as neuropathy can range from minor sensory changes like tingling or numbness to more severe injuries such as paralysis. 55-year-old male without significant past medical history experienced numbness, pain, and tingling in his left arm and forearm while lifting weights. An Electromyography-Nerve Conduction Study indicated multiple active root irritations on the left side at levels C5-C8. A Magnetic Resonance Image of the cervical spine showed a hyperintense signal lesion in the area of the cervical spinal cord from C2-C5 vertebrae, ruling out cervical myelitis. His symptoms gradually improved and were relieved for approximately 5 months with minimal signs, but recurred during daily activities. This second episode has lasted 12 months with ongoing treatment. After 12 months of follow-up, his issue has diminished but remains unresolved. It is uncommon to observe peripheral or peripheral-like symptoms in patients with recurrent peripheral neuritis and myelitis affecting only the left upper extremity. The study was approved by the ethics committee of Shiraz University of Medical Sciences with the code of IR.Sums.Med.Rec.1403.154. The patient signed the written informed consent form to document this case and include the accompanying images. It is unusual to see peripheral or peripheral-like symptoms in patients experiencing recurrent myelitis and peripheral neuritis and specifically affecting the left upper extremity. The exacerbation of symptoms when lying down on the affected side and stretching nerves is also significant. This unique presentation may be attributed to the reduced muscle mass in the proximal muscles of the upper extremity, making the nerve roots more susceptible to damage during weightlifting.

## Introduction

Stretching a nerve more than 5% of its resting length for an extended period can result in changes dysfunction and ischemia[1]. An injury to a nerve can result in issues with muscle innervation or a loss of sensation. The majority of peripheral nerve injuries occur in the upper extremity and are typically caused by trauma. Seddon classified nerve injuries into three categories: neuropraxia, axonotmesis and neurotmesis. The mildest form of nerve injury is neuropraxia, which is characterized by focal demyelination without any damage to the continuity of the nerve. Neuropraxia typically occurs after compression or traction of a nerve. The complete recovery time for this type of injury can vary from 1 week to 6 months[2]. We report a case involving recurrent peripheral nerve stretching and root irritation. The study was approved by the ethics committee at the University of Medical Sciences with the code of IR.Sums.Med.Rec.1403.154. The patient signed the informed consent to document this case and include the accompanying images.

## Case presentation

**Fig. 1:**
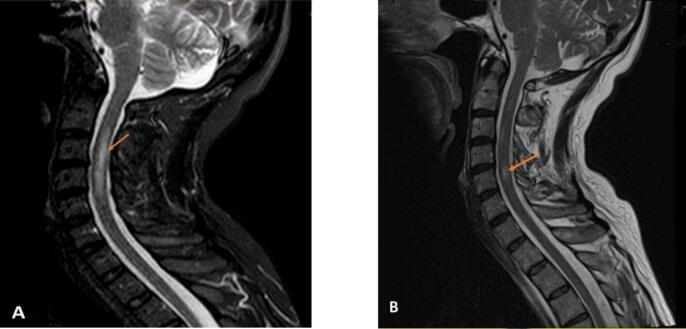
Neuroimage of the patient. A and B: T2-weighted magnetic resonance imaging (MRI). These images show hyperintense signal lesion within the C2-C5 cervical spinal cord and C5 cervical spinal cord.

A 55-year-old male (height: 165 cm, weight: 62 kg), with no significant past medical history had been in good health for the past 3 years. He experienced numbness, pain, and tingling in his left arm and forearm during exercise. Initially, he felt mild to moderate sharp and irritating pain in the left forearm, then spreading to the arm region. Over time, the patient's symptoms worsened, especially at night with increased involvement of the inner part of the arm and both sides of the forearm up to the fingertips, persisting for 5 months. He was diagnosed with peripheral Neuritis or Radiculitis due to sharp pain and the absence of Deep Tendon Reflex (DTR) in his left upper limb. There was no significant family history. Patient's physical examination showed normal muscle strength in both the proximal and distal muscles of all four limbs. However, his left upper extremity showed no response in the biceps, triceps, and brachioradialis. The patient experienced numbness, pins and needles sensation and tingling through the radial nerve and the median nerve. There was no clumsiness in the left hand; normal sensation to light touch, pin prick, and temperature was also present upon examination. Tests for Tinel's sign and Phalen's sign were negative. The deep tendon reflex was normal in the right upper limb and both lower limbs, with a downward plantar reflex. Initially, physiatrists suspected Stinger or Burner disease. Research has identified three mechanisms of burners described in the literature. The first involves a traction injury to the brachial plexus and/or nerve roots (Cervical radiculopathy). Burners often recur, with a study showing an 87% chance of recurrence. Among 36 upper level competitors who missed playing time due to a burner injury, there was a 42% chance of experiencing another neck injury resulting in the loss of playing time[3]. A first Cervical Magnetic Resonance Imaging without gadodiamide (1 month after the first episode) showed a hyperintense signal lesion in the area of the cervical spinal cord from C2-C5 vertebrae (see Fig. 1), ruling out cervical myelitis. Electromyography was performed and a brain magnetic resonance image with and without contrast was normal. The electromyography-nerve conduction study indicated multiple left-sided active root irritations at levels C5-C8. A second cervical Magnetic Resonance Imaging without gadodiamide (1 month after the second episode) revealed a hyperintense signal lesion in the cervical cord area at the C5 vertebra level (Fig. 1). His laboratory findings showed negative Neuromyelitis Optica and Myelin Oligodendrocyte Glycoprotein Antibodies and he refused to do a lumbar puncture and cerebrospinal fluid analysis. Despite receiving following mentioned treatments, the patient continued to experience numbness, pins and needles sensations, and tingling in the left arm, as well as mild to moderate stiffness in the thumb, index and middle fingers. Additionally, there was mild muscle atrophy or wasting in the dorsal and thenar eminence of the left hand during this five-month period. However, his symptoms gradually improved and were relieved for approximately 5 months with minimal signs. Unfortunately, his issue recurred while lifting an 8 kg weight bag with his left arm. He once again experienced numbness and tingling in the radial, median, and ulnar nerve areas as well as a lack of sensation on the dorsoradial side of his left hand, possibly due to myelitis extension or root entry zone involvement. Final diagnosis of the patient was recurrent Myelitis and peripheral Neuritis and due to weightlifting.

The patient was prescribed a drug regimen that included:

• Amp: Dexametasone 3 ampules 8 mg IM qod for 3 days
• NSAIDS: (Iboprufen 400 mg) po tid for 1 week
• Ointment Methyl Salisylate: associated with hot pack qd for some days
• Cap: Gabapentine: 300 mg po qd for 1 month
• Tab: Vitamin: B1 100 mg po qd for 2 weeks
• Arm Elastic Bandaging: as tolerated especially during day and night intermittently and unlimited

He experienced numbness and tingling when lying down on his left side before falling asleep, during sleep, and continuing after waking lasting throughout the day. He also felt a pins and needles sensation on the left side of his body from the neck area to the heel when he flexed his head (Lhermitte's phenomenon). Additionally, he had mild to moderate stiffness in his left thumb, index and middle fingers, as well as mild muscle atrophy or wasting in the thenar eminence of his left hand. Follow-up with the patient has, at the time of publication, shown that despite treatment, this episode has lasted for 12 months and treatment is still ongoing.

During this period, he was administered the following drugs:

• Tab: NSAIDS (Iboprufen 400 mg) po bid for weeks intermittently
• Gel: Diclofenac 1%: associated with hot pack qd some days
• Tab: Prednisolone 5 mg po qd for 10 days
• Cap: Duloxetine 20 mg po qhs for 20 days
• Cap: Pregabalin 75 mg po qd for 3 weeks
• Amp: Methylprednisolone 500 mg 3 g IV qd 1 g for consecutive 3 days
• Arm Elastic Bandaging as tolerated especially during day and night intermittently and unlimited

The patient signed the written informed consent form to document this case and include the accompanying images.

## Discussion

Most sports–related injuries involve soft tissue trauma such as ligament sprains, muscle strains, and soft tissue contusions. Cervical strains are specifically defined as stretch injuries to the musculotendinous junction[4]. When a nerve is stretched beyond its capacity, injury occurs. If the nerve is pulled beyond its limit of elasticity, the axon, myelin, and microcirculation of the nerve can be disrupted and irreversibly damaged[5,6]. Transient brachial plexopathies and radiculopathies, commonly referred to as “stingers” or “burners,” result from trauma to the brachial plexus or nerve roots[4]. Burner or stinger (acute nerve traction) is the most common sports-related peripheral nerve injury in athletes. Severe traction can lead to cervical root avulsion. When assessing an athlete with a suspected stinger, it is crucial to distinguish between a peripheral nerve injury and a central nervous system injury[5,6]. Dysfunctions in either the peripheral or central nervous system can lead to paresthesia[7]. The peripheral nervous system has a unique ability to regenerate after injury. Generally, following an injury, the peripheral nerve retains its ability to initiate regeneration for a minimum of one year. However, after severe injury, the survival of a peripheral neuron is not guaranteed[6].

## Conclusion

It is unusual to see peripheral or peripheral-like symptoms in patients experiencing recurrent myelitis and peripheral Neuritis, specifically affecting the left upper extremity. The fact that these attacks begin with weightlifting is intriguing, and the exacerbation of symptoms when lying down on the affected side and stretching the nerves is noteworthy. The presentation of recurrent episodes of myelitis with peripheral nerve signs and symptoms, but no upper motor neuron involvement during neurological examination is indeed interesting. This unique presentation may be attributed to the reduced muscle mass in the proximal muscles of the upper extremity, making the nerve roots more susceptible to damage during weightlifting. The occurrence of myelitis and radiculitis in individuals with low muscle mass, especially those prone to autoimmune disorders, raises concerns. It also suggests that myelitis could potentially stem from nerve root irritation.

## CRediT authorship contribution statement

**Abbas Rahimi Jaberi:** Supervision, Validation, Visualization, Writing – review & editing. **Ghazanfar Rafiee:** Conceptualization, Data curation, Methodology.

## Declaration of competing interest

The authors declare no conflict of interest.
